# Care pathway and prioritization of rapid testing for COVID-19 in UK hospitals: a qualitative evaluation

**DOI:** 10.1186/s12913-021-06460-x

**Published:** 2021-05-31

**Authors:** Timothy Hicks, Amanda Winter, Kile Green, Patrick Kierkegaard, D. Ashley Price, Richard Body, A. Joy Allen, Sara Graziadio, CONDOR Steering Group, D. Ashley Price, Richard Body, A. Joy Allen

**Affiliations:** 1 NIHR Newcastle In Vitro Diagnostics Co-Operative, The Medical School, Newcastle University, Newcastle upon Tyne, NE2 4HH UK; 2 The Newcastle Hospitals NHS Foundation Trust, Royal Victoria Infirmary, Newcastle Upon Tyne, NE1 4LP UK; 3 Translational and Clinical Research Institute, Newcastle University, Newcastle upon Tyne, NE1 7RU UK; 4 NIHR London In Vitro Diagnostics Co-operative, Department of Surgery and Cancer, Faculty of Medicine, Imperial College London, London, UK; 5 CRUK Convergence Science Centre, Institute of Cancer Research & Imperial College London, London, UK; 6 Division of Cardiovascular Sciences, The University of Manchester, Manchester, M13 9PL UK; 7 Emergency Department, Manchester Royal Infirmary, Manchester University NHS Foundation Trust, Manchester, M13 9WL UK

## Abstract

**Objectives:**

The second wave of the coronavirus pandemic is now established, occurring at a time of winter pressure on acute care in the NHS. This is likely to be more challenging then the first wave for the diagnosis of COVID-19 because of the similar symptomology with other respiratory conditions highly prevalent in winter. This study sought to understand the care pathways in place in UK NHS hospitals during the first wave (March–July 2020) for identification of patients with COVID-19 and to learn lessons to inform optimal testing strategies within the COVID-19 National Diagnostic Research and Evaluation Platform (CONDOR).

**Design, setting & participants:**

Sixteen hospital-based clinicians from 12 UK NHS Trusts covering 10 different specialties were interviewed following a semi-structured topic guide. Data were coded soon after the interviews and analysed thematically.

**Results:**

We developed a diagrammatic, high-level visualisation of the care pathway describing the main clinical decisions associated with the diagnosis and management of patients with suspected COVID-19. COVID-19 testing influenced infection control considerations more so than treatment decisions. Two main features of service provision influenced the patient management significantly: access to rapid laboratory testing and the number of single occupancy rooms. If time to return of result was greater than 24 h, patients with a presumptive diagnosis would often be cohorted based on clinical suspicion alone. Undetected COVID-19 during this time could therefore lead to an increased risk of viral transmission.

**Conclusions:**

During the winter months, priority for provision of rapid testing at admission should be given to hospitals with limited access to laboratory services and single room availability. Access to rapid testing is essential for urgent decisions related to emergency surgery, maternity services and organ transplant. The pathway and prioritization of need will inform the economic modelling, clinical evaluations, and implementation of new clinical tests in UK.

**Supplementary Information:**

The online version contains supplementary material available at 10.1186/s12913-021-06460-x.

## Strengths and limitations

- Three methodologists trained in care pathway analysis and IVD evaluation developed a topic guide and draft pathway in collaboration with two clinicians who specialise in emergency medicine and infectious diseases.
- Overall, 16 clinicians were interviewed, covering 10 clinical areas from 12 Trusts across the UK in order to understand the care pathway for COVID-19, the diagnostic challenges during the first wave, lessons learnt that can be useful for the winter season, and the prioritisation of patients for testing.
- The sample size was relatively small: saturation of information was reached quickly even though the sample was varied, possibly due to strict, mandatory regulations facilitating a common approach across the UK.
- The interviews were analysed thematically, and the results delivered at pace, in order to directly inform clinicians, policy makers, researchers, modellers, and regulators.
- Patients and the public were consulted and felt this work was of national importance. Their feedback informed the discussion and the assessment of value of the research and the dissemination of the research findings.

## Introduction

The global SARS-CoV-2 pandemic has put considerable pressure on healthcare services worldwide [1]. Health and care providers require clear care pathways and diagnostic strategies to rapidly identify patients infected with SARS-CoV-2, to ensure appropriate use of single patient occupancy side rooms, accurate cohorting of patients and early administration of therapies for coronavirus disease (COVID-19) [2]. The risks of nosocomial infection are high [3] and mechanisms to limit transmission of SARS-CoV-2 within hospitals and care facilities are an urgent priority, particularly as many countries are now dealing with a second wave of infection [4].

In the UK, as the second wave is coinciding with the winter months, clinicians are faced with further complexity in the diagnosis of COVID-19 due to the similar symptomology with other viral respiratory illnesses [5, 6]. Ensuring a detailed understanding of identification and management strategies adopted during the first wave of infections is crucial in order to inform the design of effective clinical pathways for admitted patients. There is pressure to expedite test development and evaluation, and the demand on developing models to help identify optimal testing strategies is also high. Patient care pathway analysis [7] is the first step in that direction, supporting the identification of the most effective role of new tests in the current care pathway [8].

The aim of this work was to articulate at a high-level, the UK NHS hospital care pathway for patients with suspected COVID-19, focusing on testing strategies during the epidemic in May and June 2020. This work is the first of this kind and informed the National Institute for Health and Care Excellence (NICE) early economic modelling exercise to evaluate options for testing strategies in the winter season when multiple respiratory conditions with similar symptomatology are circulating. This work also informed the clinical study planning within the COVID-19 National Diagnostic Research and Evaluation Platform (CONDOR) (https://www.condor-platform.org/), which is currently evaluating new diagnostic tests to support the early identification of COVID-19 in hospitals and in the community.

## Methods

### Design

A qualitative study, using semi-structured, in-depth videoconferencing interviews and thematic analysis was carried out. Data were collected in 13 hospital sites within 12 NHS Trusts located in England, UK. The COREQ-checklist [9] and Standards for Reporting Qualitative Research (SRQR) guidelines [10] were followed to ensure study rigour, trustworthiness and transparent reporting.

The project was approved by the research office of the Newcastle upon Tyne Hospitals and registered as a service evaluation on the Trust records (Project no. 10222).

### Recruitment and setting

This UK-based service evaluation aimed to recruit a sample of clinicians working in secondary care organizations who were in frequent interaction with patients with suspected or confirmed COVID-19. Purposive sampling was used to select participants to include a range of years of experience and those who were likely to be knowledgeable of pathways and procedures throughout the pandemic. Identification and recommendations for interviewees were made from existing networks of clinicians, e.g. the CONDOR platform steering group (https://www.condor-platform.org/) and associated organisations.

The primary characteristic of those who were approached for interview were physicians working within: Emergency Medicine, Acute & General Medicine, Respiratory Medicine, Intensive Care, Infectious Diseases, Microbiology, and Virology. Throughout the interviews, secondary specialisations which were felt to be important were identified, and further professionals were recruited using snowball sampling; to reduce regional bias additional clinicians from a previously interviewed trust were not approached during the snowball sampling.

Between June and August 2020, 26 clinicians were invited by email to participate in the study, 16 of which eventually took part in the study and 10 declined or did not respond. No clinicians who agreed to take part in the study subsequently withdrew. Clinicians received an information sheet and were offered the opportunity to discuss the study prior to interview. Recruitment to the study was concurrent with data analysis, and data collection proceeded until no further unique themes were identified in successive interviews (saturation) [11, 12]. Data saturation, meaning that no new codes emerged from the analysis, was reached after 14 interviews. Two additional interviews were performed in which data saturation was confirmed.

### The topic guide

The protocol for the evaluation and a topic guide were prepared by a Senior Clinical Test Evaluation Methodologist (SG) and two Clinical Test Evaluation Methodologists (TH and AW) trained in care pathway analysis from the NIHR Newcastle In Vitro Diagnostic Co-operative (who also conducted the interviews). Included in the topic guide was a test case priority table designed by the methodologists, to elicit key use cases, patient priorities and key characteristics of the test. The topic guide was piloted first with two clinicians working in infectious diseases, these were not included in the analysis. The interviews were semi-structured and covered questions about the care pathway for COVID-19, the diagnostic challenges during the first wave, lesson learnt that could be useful for the winter season and prioritization of patients for testing.

A draft care pathway diagram was produced from preliminary discussions with clinicians from The Newcastle Hospitals NHS Foundation Trust and Central Manchester University Hospitals NHS Foundation Trust working in Infectious Diseases and Emergency Medicine respectively. The pathway was validated through the interviews, also capturing variability of care across the UK.

### Data collection

The topic guide and care pathway flow diagram (see supplementary material) were all sent to the participants at least 12 hrs ahead of the interview taking place so that they were able to familiarise themselves. Topic guides included key questions, with probes and prompts used where appropriate to allow for flexibility and to ensure that participants had the opportunity to discuss subjects they deemed important.

Interviews were conducted via videoconferencing software, either Microsoft Teams (Microsoft Corporation, Redmond, WA, USA) or Zoom (Zoom Video Communications, Inc.), dependant on participant preference, either whilst the participant was at home or their place of work. All interviews were led by one of the research team’s researchers with qualitative training (TH, SG and AW). The interviewers were not previously known to 13/16 of the participants, and 3/16 were already familiar with at least one of the interviewing team. Only the interviewee and the researchers were present in the video call, however, some clinicians who were at their place of work were using shared office space with others present albeit not actively participating. The interviews were scheduled to last 1 h, and no interviews were repeated. Interviews lasted from 49 to 102 min (mean 65 min). Participants were not compensated for participation.

The interviews were recorded (if consent to do so was given by the participant), and detailed field notes were taken during the interviews. Due to time constraints, notes were not returned to the participants for comment or correction.

### Data analysis

Audio recordings were extracted from the initial video recordings before being anonymised, with all identifying information removed or replaced with pseudonyms. Detailed field notes taken during the interviews were verified against audio recordings by an investigator who did not compile the original notation (either TH, AW or KG). Data management was facilitated using an Excel spreadsheet. All data were collected, entered and then analysed by two investigators (TH and AW). Initial coding was carried out as soon as practicably possible by AW or TH.

Using methods of thematic analysis, both investigators independently listened to the audio recording and read the notes, coding the data deductively [13, 14]. Using content analysis approach, the researchers sorted the coded data into predefined themes determined by the research questions [13, 14]. This coding was then verified by SG at a later date.

As a whole, this process enabled identification of the main factors, including both diagnostic and logistical, driving the choice of cohorting pathways for patients in secondary care during the SARS-CoV-2 pandemic. In this article we present these findings, where illustrative quotes are used to represent a range of points of view and backup the findings. To protect participant anonymity, participants are referred to only by number, P1-P16.

### Public and patient involvement and engagement panel

Following on from the interviews with the clinicians, the pathway and thematic summaries were presented to the NIHR Newcastle MIC Insight Panel [15] comprising of 5 members of the public (3 male and 2 female) with a keen interest in healthcare. Members had a wide range of backgrounds and careers, with different experiences across multiple sectors. Members were asked for their opinion about the pathway and their views as to how diagnostic testing in secondary care may be utilised.

## Results

### Clinician characteristics

The 16 clinicians interviewed (14 Male, 2 Female) were all working in the NHS during the pandemic. Eleven specialisms were covered by the clinicians, these being: Acute Medicine, Critical/Intensive Care, Emergency Medicine, Gastroenterology, General Medicine, General Surgery, Infectious Diseases, Microbiology, Nephrology, Respiratory Medicine, and Virology. The clinicians had a median post qualification experience of 25.5 years (Range: 10–32 years) and covered 12 NHS trusts with a median size of 801 beds (Range: 327–1454). Eleven clinicians reported having on-site laboratories performing SARS-CoV-2 testing, with 5 reporting their laboratories were offsite.

### Narrative summary of pathway

#### Infection prevention

**Fig. 1:**
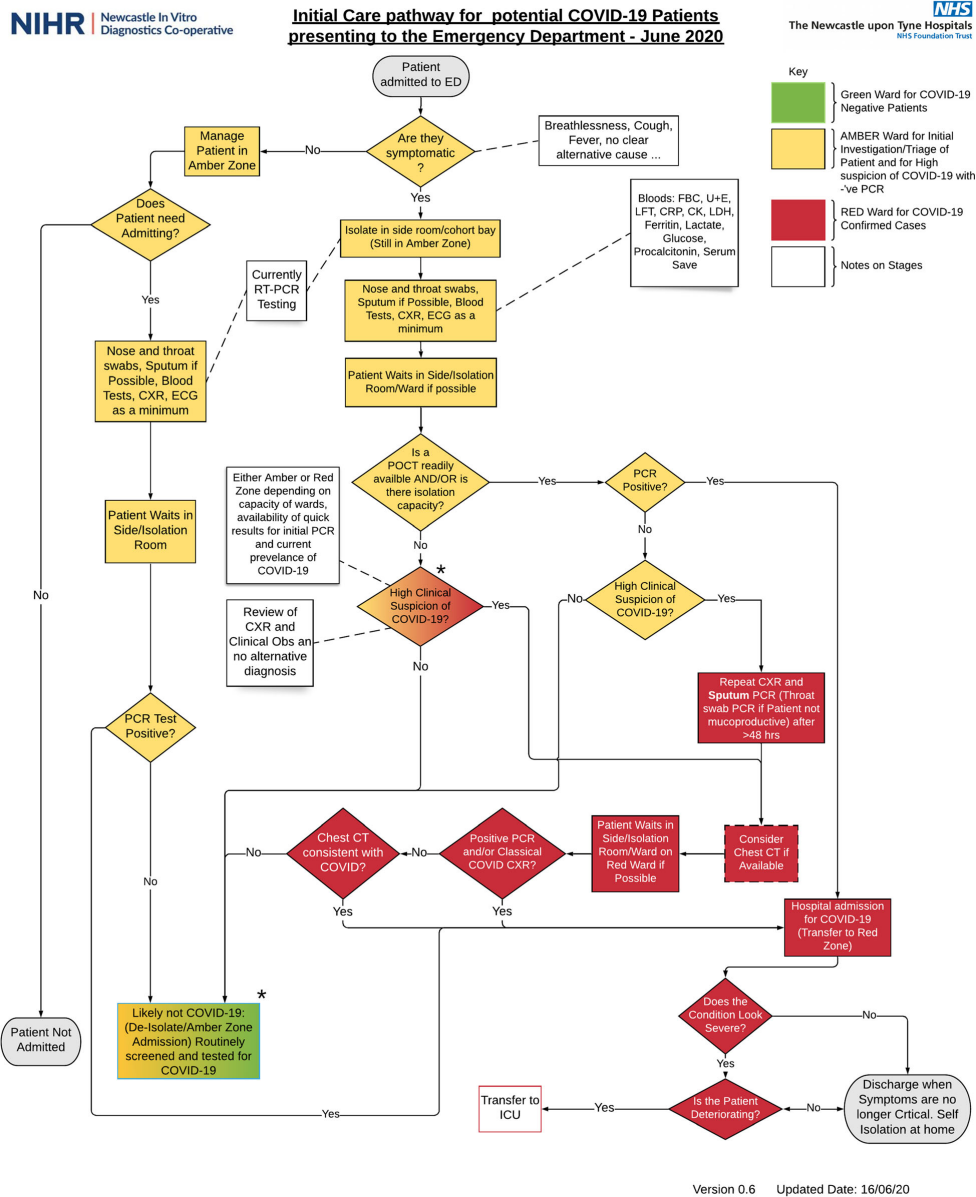
Initial care pathway developed through a combination of national guidance and expert opinion before being presented to the interviewees. Patient areas are designated as one of three zones: Red, Amber, and Green depending on the clinical suspicion or diagnosis of COVID-19. Early triage of initial symptoms determines whether the patient is isolated prior to additional tests being ordered, or cohorted together in a separate area. Only when a positive SARS-CoV-2 test is returned, or a clinical diagnosis of COVID-19 given following additional tests, e.g. chest X-ray, is the patient transferred to a Red zone. *Indicates areas in which, due to the external prevalence, the zoning criteria may change. In these cases, with a high prevalence the decision to assign a patient to a Red zone may be taken on clinical suspicion alone to improve patient flow. Additionally, with a high prevalence a single negative SARS-CoV-2 result may not be enough to justify admission into a Green zone

**Fig. 2:**
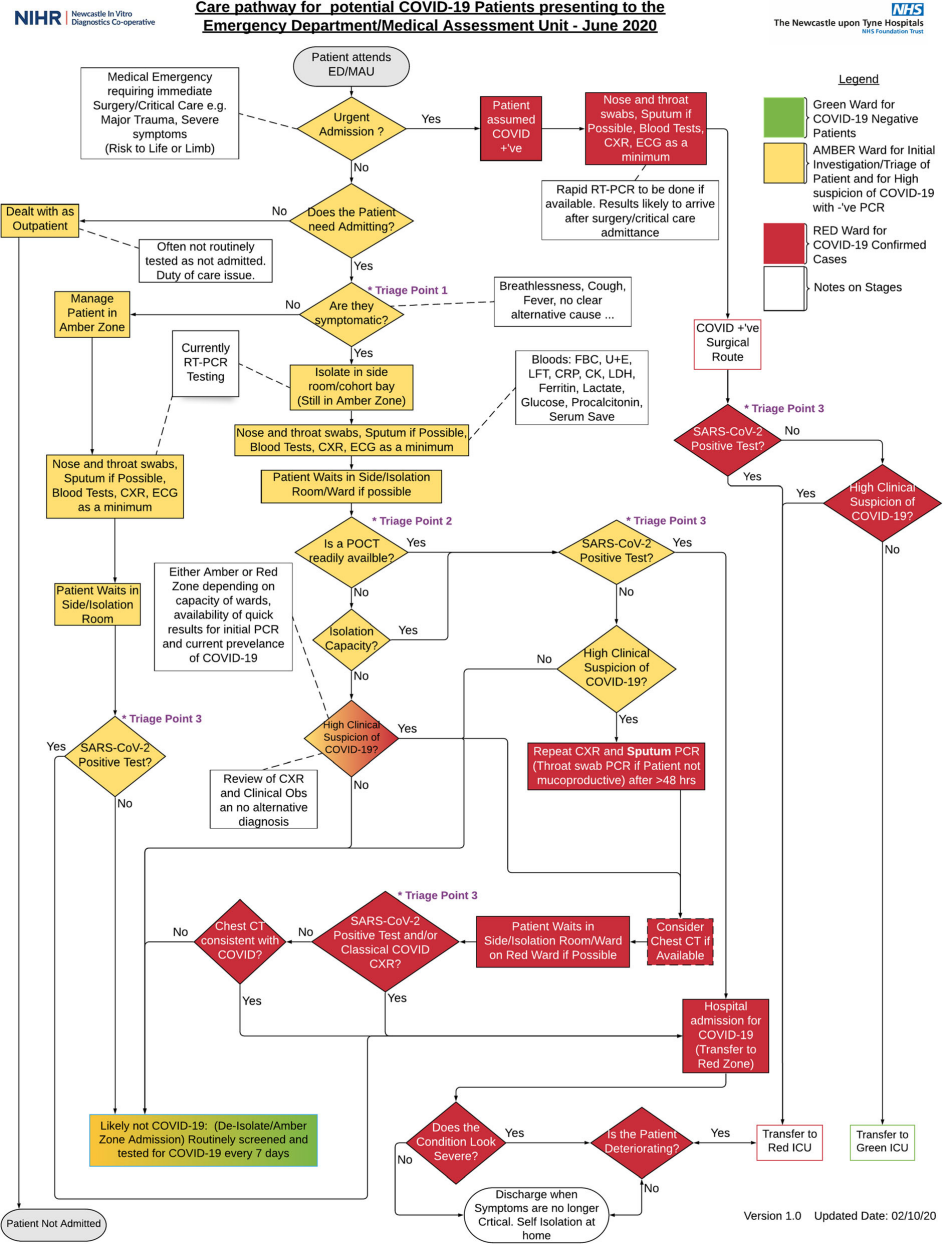
The revised patient pathway constructed following consultation with the clinicians. Additional triage points (Indicated as Triage Point 1, 2, and 3) have been included to show stages within the pathway where testing and clinical observations are routinely performed. Additional stages have been added following Triage Point 2 to highlight the limiting factors of real-estate/isolation capacity and the availability of rapid/POC testing. If these factors are limited, the decision to cohort can be based purely on Clinical Suspicion (Centre diamond in Red/Yellow). An additional surgical pathway (shown on the right) has been added to highlight the assumption of SARS-CoV-2 positivity due to the urgency of the admission

Most interviewees agreed that the initial draft pathway (presented in Fig. 1) which was presented to them was representative of both the peak of the first pandemic wave (April 2020) and the following reduction in prevalence observed in June with either minimal or no changes being required. The interviews resulted in 3 different iterations of the draft pathway in order to capture additional areas or adjust some diagnostic stages, with the final pathway providing a general overview of admissions into secondary care (presented in Fig. 2). The focal point of all the pathways was related to patient management, infection control, and improving diagnostic detection. Discussions around individual therapeutic options at each stage were not investigated.

Whilst there was a small difference in the approach to isolation and cohorting across different trusts, predominantly due to real-estate and staff availability, the approach used by the majority of interviewees was to cohort patients into areas based on the result of a COVID-19 diagnosis. In general, these areas can be split into 3 categories: **Green**, **Yellow**, and **Red**.

**Green** - Negative COVID-19 diagnosis

**Yellow** – Pending COVID-19 diagnosis

**Red** – Positive COVID-19 diagnosis

**Fig. 3:**
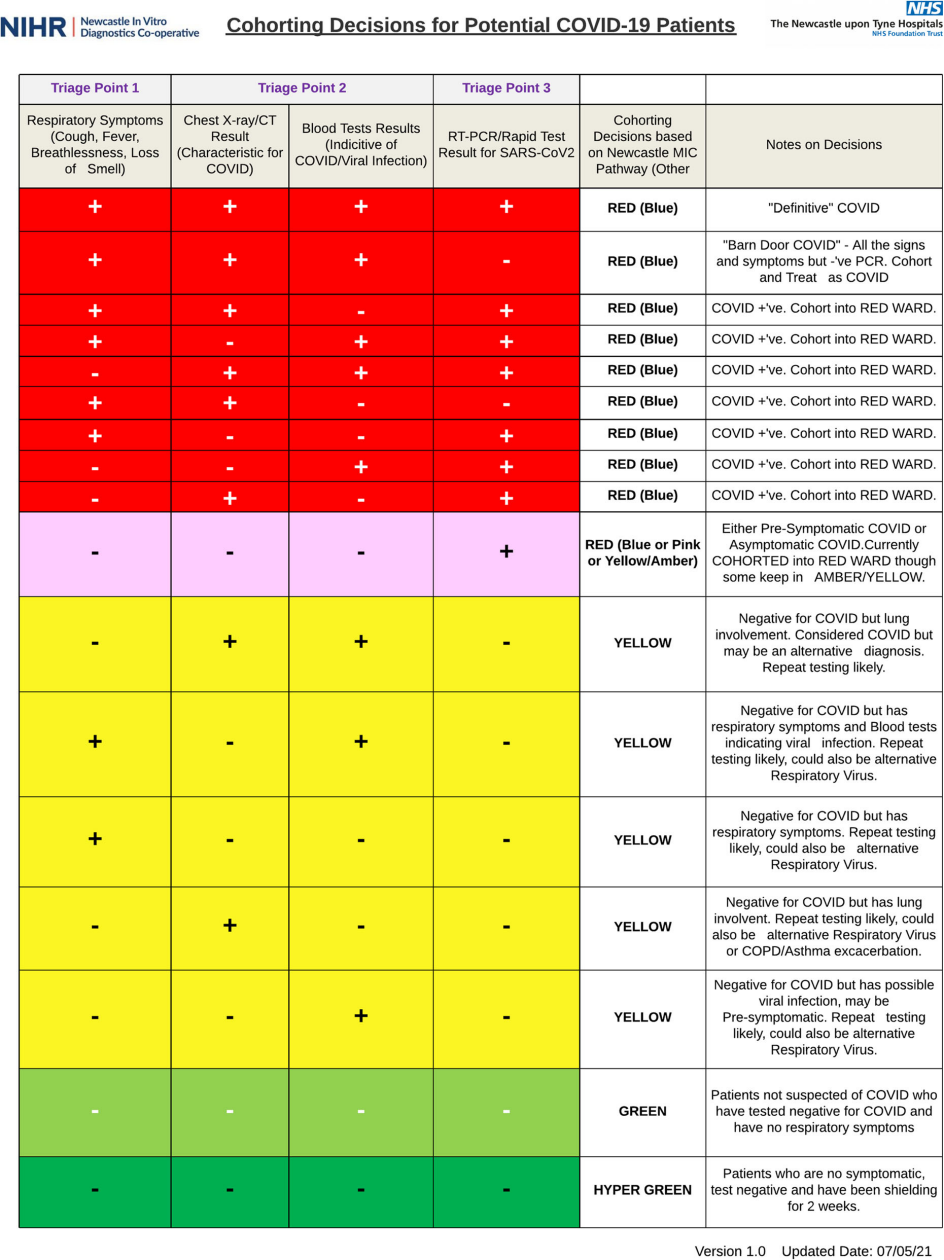
The determining factors in diagnosing COVID-19 based on test results, presenting symptoms, clinical observations and imaging. Each Triage Point denotes a separate time point in which the results of the testing would be determined. Following the results, the patients were cohorted accordingly into Red, Pink, Yellow, Green, or Hyper Green zones. Zones donated as a different colour, i.e. Blue, Yellow/Amber, indicate a difference in cohort terminology across NHS trusts. Common blood tests results for viral and respiratory infection used as a proxy for COVID-19 diagnosis during the first wave were: a full blood count showing leukocytopoenia, lymphocytopoenia and neutropoenia [16]. Thrombocytopoenia was also sometimes used by clinicians as an indicator of COVID due to it not being a common finding in infection

During the pandemic, a positive COVID-19 diagnosis was determined through a combination of clinical observations, blood tests, imaging, and Real time reverse transcriptase - polymerase chain reaction (RT-PCR) (See Fig. 3.). Therefore, a positive COVID-19 was described as either:

- A positive SARS-CoV-2 test
- A clinical diagnosis based on symptoms/X-Ray/Computed Tomography (CT)

The initial stage for most hospital pathways was to determine if the patient needed admitting into secondary care. Any patients not needing a hospital admission after the initial assessment in the Emergency Department (ED), were not routinely tested for SARS-CoV-2, however one clinician did highlight that this could be a failure in the duty of care as:

*“At the moment we don't test you if you go home. That's a policy thing. Personally, I think if you come in with respiratory symptoms, I think we should test you. So that then we can do track and trace in the community, from a public health point of view. And so I think what we're doing at the moment is wrong, but that's what we're doing.”* **P10**

Any patients who needed urgent surgical admission, with a threat to life or limb, requiring intensive care unit (ICU) admission were assumed to be SARS-CoV-2 positive in order to protect the staff and ensure the correct infection prevention protocols were followed with regards to personal protective equipment (PPE) and an enhanced cleaning regime. These surgical patients were then cohorted following the result of a swab taken pre-surgery, with positive patients being transferred to a **Red** ICU and negative patients being transferred to a **Green** ICU.

Any non-urgent medical/surgical patients were triaged based on the presence of respiratory symptoms (including cough, fever, and anosmia/ageusia). This initial triage often took place in the ED or within the Medical Assessment Unit (MAU), which was deemed as a **Yellow** zone. Within this zone those presenting with respiratory symptoms were isolated into individual side rooms, where possible, or else cohorted together in a bay with other respiratory patients. Whilst those presenting without respiratory symptoms were cohorted together in another bay or section of the department.

Patients were then held in a **Yellow** zone until a result from a SARS-CoV-2 test was available. Where the result of the test differed from a high clinical suspicion (severe symptoms, blood results, and X-ray/CT indications) a second test was often ordered due to the high potential for false negatives. Centres differed on their approach to these patients, with some centres transferring them out of the ED/ MAU and into a **Yellow** back of house ward. This was because the high turnaround time for testing and result reporting (4–12 h with an in-house lab, 12–24 h if externally tested) would have otherwise resulted in a bottleneck at the ED/MAU stage.

The main factors in determining the patient route therefore were real estate capacity, testing time to result, the number of patients presenting, and the current prevalence of SARS-CoV-2 in the local population.

#### Areas of deviation from the typical pathway

##### Testing criteria

During the initial peak of the pandemic (March–April) the majority of trusts interviewed reported that they were only testing patients for which COVID-19 was clinically suspected. As the situation evolved however, all admitted patients were then tested for SARS-CoV-2 upon admission and routinely tested once admitted to a ward to identify nosocomial infection, with all trusts following this procedure by the beginning of June.

##### Cohorting

A few trusts developed additional cohorting zones and modified existing ones beyond the standard **Green**, **Yellow**, and **Red**. These trusts were often those with a larger amount of capacity and real estate. Additional zones (see Fig. 3) across the different trusts were:

- AMBER: This zone was used in trusts where the clinical decision making was aided by the SARS-CoV-2 diagnostic test results. In this case there was a high clinical suspicion of COVID-19 based on the symptoms, but this alone wasn’t enough to justify moving to a **Red** zone and instead the clinician would wait on a test results before moving the patient to **Red** or **Green** (rarely; only if bed space was critical).
- YELLOW (Modified): Clinicians used this zone where COVID-19 could not be excluded, until a SARS-CoV-2 test result was returned, even though there was a low clinical suspicion of COVID-19. The subsequent cohorting and patient management depended on the test result.
- PINK: Patients who had a positive test result for SARS-CoV-2 but no respiratory symptoms were designated as PINK. This group included both asymptomatic and pre-symptomatic patients. Those who then developed symptoms were transferred to a Red Ward.

Two trusts reported not using a **Yellow** ward for one of two reasons. The first was due to a high prevalence and restrictions in real estate leading to a triage outside of the hospital (P14), whilst the second did not have a **Yellow** zone due to disagreements between clinical opinion concerning clinical diagnosis with a negative swab (P7). To avoid confusion as to the zoning, only patients with a positive SARS-CoV-2 test result were sent to the **Red** wards.

### Major themes

Analysis of the interviews generated 5 key themes with regards to SARS-CoV-2 testing and its role in secondary care (Shown in Table 1).

**Table 1:** Major Themes

***Key Themes***	***Description***	***Example Quote***
**Decision making Problems**	Problems highlighted with regards to patient flow, infection prevention and zoning classifications	“*We couldn’t keep them in the respiratory assessment unit because there was a flow of patients backing up in ED”* ***P11***
**Testing**	Current tests in use by trusts, along with considerations regarding accuracy, availability, type of test and routine screening	*“we’ve got access in ED to 20 rapid tests per day [which] may come back in a couple of hours...And we try and reserve those for patients who are in particular patients who are going for urgent surgery … And the PCR will come back in several hours or possibly the next day”* **P10**
**Next Winter Season**	Expected complications of diagnosing COVID-19 during the next winter season and the role and perceived use of differential testing	*“...when we get to the winter, it can be extremely difficult to triage people. Because … clinically, they look the same...they will all have a temperature, they will all have a cough, some will have chest X ray changes, some won’t, and being able to cohort these people will be more difficult.”* **P7**
**Future Testing**	The expected characteristics needed in future testing, along with priorities for testing in the event of a limitation in testing availability	“*… in terms of you asking about point of care, it has to be interfaced with our laboratory with our laboratory information management system, and EPR [Electronic Patient Record].”* **P2**
**Lessons Learned**	Key lessons learned from the first wave, both positive and negative.	*“[We were] using an expert panel to filter the cacophony of noise into something which was decent quality, and also had usefulness. And I think managing information flow … was probably the best thing that we did locally.”* **P10**

### Problems with decision making

A large problem highlighted by the clinicians was that the **Yellow** and **Red** zones were essentially ‘real-estate’ on loan from elective departments (routinely orthopaedics) and with the drive to reopen elective procedures, zone sizes could diminish as prevalence increases during a second wave.

The majority of clinicians thought therefore that it would prove difficult to achieve the correct balance of **Yellow** and **Red** zones needed at any given time and that modelling would be useful to help understand when to adjust the ward levels for COVID-19 based on external prevalence. Additionally, it was noted that reducing the zones and limiting patient flow could result in infection control issues when an individual within a cohorted area returned a positive result and the others didn’t.

A common trend across sites was to also designate patients as *“always* ***Red****”* during a hospital admission, with patients not being moved from a **Red** zone into a **Green** zone, even with no symptoms and/or a negative swab unless capacity was critical.

Clinicians also regularly highlighted that, whilst patients within the **Green** zone were regularly screened for SARS-CoV-2, staff members were not. This then made the **Green** status difficult to maintain without regular staff testing, ignoring a route of nosocomial infection. Due to this, one hospital (P11) took the approach of treating their **Green** areas as an extension of their **Yellow** areas.

#### Elective pathways

Elective surgical pathways took a different approach to admission, often being called **Hyper Green** and based within a separate hospital, where possible. Whilst there was some variation between regions, the testing and isolation regime was very similar. Patients would self-isolate at home for 14 days prior to their surgical date, with a swab being taken 3 days before their surgery. Any patient whose SARS-CoV-2 result came back positive had their surgery cancelled, as did any who presented with respiratory symptoms on the day.

### Testing

#### Availability

Real time RT-PCR based laboratory tests were available for all patients who required admission to hospital, across centres. Testing was generally on combined throat/nose swab due to availability of swabs, but sometimes individual nose and throat swabs were used. The turnaround time for RT-PCR varied significantly with a range of 3-48 h. Turnaround time was very slow at the start of the pandemic dues to limited number of Public Health England (PHE) facilities providing tests and the limited availability of reagents.

*“Sometimes we waited for three days for the test to come in the early days. So we were left in area of uncertainty based on just clinical presentation.”* **P1**

*“As an organisation we didn’t have testing in-house until very late on, which hampered our response. So we had to have a plan which didn’t rely on testing, because we were waiting 36-48hrs for test [results] to come back.”* **P5**

The turnaround time improved over time as more centres moved to in house testing, but some were noticing that this had since slowed again due to reagent shortage, the need to batch tests in some centres to conserve resources and the increased demand for testing from elective pathways (referring to the month of July when elective admissions began again). The unreliable, centralised supply of regents led a number of trusts to diversify their testing platforms i.e. using more than one, in an effort to ensure testing was always available.

#### Accuracy

The accuracy of RT-PCR was highlighted as a problem, especially for the initial PHE tests. The rate of false negatives was reported as high by clinicians, resulting in re-testing (and/or ignoring a negative test result), when COVID-19 was highly clinically suspected.

*“ … the Public Health England test that have really high false negative rate. We thought maybe it was only about 60% sensitive and in the end was quite difficult.”* **P15**

#### Rapid/point of care testing

Some centres reported either using rapid testing systems, defined as platforms within the laboratory which can provide a result in a couple of hours regardless of whether they use antigen detection or viral amplification, or a point of care (POC) test, with POC tests being those typically used outside of the laboratory and with a short time to results.

Those centres with current rapid testing systems, such as the Cepheid Gene Xpert [17], often stated that the rapid turn-around of the test was ideal for urgent admissions; however, the lack of reagent availability meant that these tests were not being fully utilised. One participant commented that, as their trust had an allocation of five rapid tests a day, they moved away from the platform all together.

*“We had a very rapid one [Cepheid], that we had very tiny numbers [5/day] for, that actually it’s become more trouble dealing with the requests and monitoring requests for this small number of rapid response tests. We’ve kind of abandoned that one.”* **P15**

The use of these tests was restricted to managing urgent surgical, interventional and bed management situations and to support transplantation programs. An approval or triaging system was often in place to ration the usage of these tests.

One trust was using an experimental POC test outside of the laboratory in ED and theatres, which was performed by nursing staff. All others placed their rapid/POC testing within their laboratories.

#### Staff testing

The provision of staff testing was not consistent across organisations. Most provided RT-PCR testing to symptomatic staff only; others provided weekly or fortnightly testing to asymptomatic staff who treated high risk patients or work exclusively in green zones. Some felt that the major component of maintaining green zones was to prevent transmission from staff.

*“It doesn’t seem to me that there is any clear guidance on what trusts should be doing, and as a result it’s a bit mix and match. Where programs have started it’s generally for staff who are looking after what would be considered to be more vulnerable patients.”* **P8**

*“That’s why, in my mind, we can’t consider ourselves to be green, because we are not testing staff...we don’t have once or twice weekly testing to pick up the asymptomatic infectious staff.”* **P11**

Most had been offered antibody testing at their workplace but were unsure of the significance of the result outside of epidemiological interest.

#### Antibody testing

All participants in the study commented that antibody tests in their current state and level of knowledge, demonstrated no added value to acute patient care pathways. The main utility of antibody tests currently is showing incidence and assessing the number of asymptomatic individuals.

Although there was strong sentiment that the basic science of what seroconversion means in terms of immunity needed to be better understood before it could be of any use clinically, some participants did state that antibody tests could be useful as part of a composite diagnostic strategy.

*“I’ve got a patient who’s got a pneumonitis on chest x-ray (ground glass) and confirmed on CT, but not typical of COVID … So now she’s swab negative, fine. So now it comes back with antibodies … So if she’s swab negative, antibodies negative and CT equivocal, I can move her into a green area.”* **P7**

### Concerns about the next winter season

#### Expected complexity

The interviewees felt that it was “easier” to diagnose COVID-19 during the first wave of pandemic, as there were few other respiratory viruses co-circulating at the time. All agreed that it would be extremely difficult to distinguish COVID-19 from many other respiratory viruses (including Influenza (Flu) A/B and Respiratory Syncytial Virus (RSV)) in the coming winter season.

*“I think this winter’s going to be very interesting. Syndromically, you can't really distinguish COVID-19 from influenza reliably, or indeed potentially other severe respiratory tract infections that might be presenting in the winter, such as RSV or even parainfluenza.”* **P6**

The model which was adopted for the first wave of the pandemic will not be suitable for any subsequent waves, as capacity was ‘borrowed’ from elective pathways which had ceased activity. These pathways need to find a way to continue to operate in future or else secondary deaths will rise.

*“The system that we had in place [for the first wave], I think worked reasonably well. But I think it worked well because there was nothing else going on in the hospital. So actually, we always had lots of empty beds, which is something we never normally have, so we had a good amount of capacity. We were very well staffed because all staffing levels were kind of doubled. When there was lots of COVID then actually a lot of these people were presenting pretty sick with a pretty typical presentation … I think we're dealing with a totally different situation now and I think almost nothing from the first wave will be applicable to this coming winter unless we have another really big wave of COVID.”* **P4**

Capacity and cohorting is likely to be increasingly difficult, with isolation capacity and side room availability put under pressure not only by SARS-CoV-2 but also other infections such as norovirus. Co-infection with multiple pathogens is not improbable and this could be challenging from both a diagnostic and clinical management perspective.

#### Multiplex testing

Clinicians generally agreed that multiplex testing, i.e. a test which targets multiple pathogens on one assay, was needed and useful to facilitate isolation strategies during the winter. The primary utility would be for infection prevention and control, enabling effective cohorting to reduce cross infection. The results might have a direct influence on treatment options, such as cessation of antibiotics, the selection of appropriate treatment option or identifying patients who might be safely discharged. Some felt that most treatment of respiratory viral illness was managing symptoms and that distinguishing between viral aetiologies may not help from a treatment perspective.

*“I think we desperately need them to be multiplexed for the lab, for the lab assays, and probably also for point of care tests … … so that we can just have a standard respiratory PCR test where you get COVID, flu, potentially RSV … ..all in the same in the same test, because otherwise, there's a lot of duplication of effort from the lab staff of having to process [tests] for several different things.”* **P4**

Use of Flu A/B POC testing had been in widespread use last winter and was generally positively received. Some were not using Flu POC tests due to biosafety concerns. Opinion was divided as to whether RSV would be needed on a multiplex panel. This was dependent on clinician background with microbiologists more inclined to a full panel than clinicians working in emergency departments. RSV would be important in some populations such as paediatrics, the older and those with chronic obstructive pulmonary disease. Minimal specification for a multiplex test should be Flu A/B and SARS-CoV-2, but the inclusion of RSV may be beneficial. Additional inclusion of other Flu subgroups and common respiratory viruses may also be useful.

*“ … our winter planning certainly for the lab is only for tests that will test for Flu [and] RSV at the same time as COVID.”* **P2**

*“I’m not going to use RSV in a point of care combination for adults because there is no infection issue.”* **P9**

### Future testing

#### Use case priorities

Using the test case priority table presented to them as part of the topic guide, the clinicians highlighted patient groups who they felt should be prioritised in getting a rapid or POC test, focusing on risk categories of patients and severity of symptoms, in the event of limited resources. Clinicians also commented on whether they felt a high sensitivity or specificity should be the priority in test development. Additionally, the clinicians reported on use cases which would benefit the most from having rapid or POC testing. The results of this discussion are presented in Table 2 below:

**Table 2:** Use Case Priorities

ID	Specialty	Test Priority (Symptoms, Risk)	Sensitivity or Specificity Priority	Thoughts on Use Case Priorities
**P1**	Critical Care	N/A	N/A	Elective Surgery, Differential Cohorting, Patient Flow, Urgent Surgery
**P2**	Microbiology and Pathology	Mild Symptoms, Asymptomatic	N/A^I^	Elective Surgery, Patient Flow, Urgent Surgery
**P3**	Emergency Medicine	Severe/Mild Symptoms, Asymptomatic (High Risk)	Sensitivity	Elective Surgery, Patient Flow, Urgent Surgery, Dialysis, Chemotherapy, Staff Testing, AGP^a^,
**P4**	Infectious Diseases	N/A	N/A	Differential cohorting, Patient Flow
**P5**	Emergency Medicine and Acute Medicine	N/A	Sensitivity	Clinician Dependent
**P6**	Virology and Microbiology	Severe/Mild Symptoms	Specificity	Urgent Surgery, Transplant
**P7**	Acute Respiratory	Shielding Patients	N/A	AGP, Patient Flow
**P8**	Infectious Diseases	Mild and Asymptomatic	N/A	Emergency and Acute Admissions, Maternity
**P9**	Microbiology and Infection Control	N/A	Sensitivity	Elective Surgery, Patient Flow
**P10**	Emergency Medicine	Mild/Unclear Symptoms	N/A	Dialysis, Urgent Surgery
**P11**	Gastroenterology and General Medicine	Mild Symptoms	N/A	Patient Flow, Respiratory ED, Surgery
**P12**	Nephrology	N/A	N/A	Elective Surgery, Patient Flow, Transplant
**P13**	General Surgery	Mild/Unclear Symptomatic, Asymptomatic	Sensitivity	Chemotherapy, Surgery
**P14**	Acute Medicine	Mild/Unclear Symptomatic, Asymptomatic	Specificity	AGP, NIV^b^, Patient Flow
**P15**	Acute Medicine	N/A	Sensitivity	Maternity, General Anaesthetics, NIV, Endoscopy
**P16**	Retinal Surgery	High Risk Patients	N/A	General Anaesthetic

N/A - The interviewee was unsure, didn’t explicitly state a priority, or wasn’t explicitly asked

I The Clinician commented that the potential for user error was having more of an effect than any low sensitivity/specificity

^a^Aerosol Generating Procedures (AGP), ^b^Non-Invasive Ventilation (NIV)

The major priority for the placement of rapid testing was in areas where delay had an impact on patient flow and/or infection prevention and control. Patients requiring aerosol generating procedures needed rapid testing to protect staff and other patients. Patients who required a general anaesthetic also required testing as outcomes for COVID-19 positive patients was poor. How rapidly this testing needed to be performed depended on the urgency of the procedure.

*“In the ED what we are fundamentally managing is time … an additional one hour wait in the department results in a 30% drop in cubicle efficiency, which is huge. [The time to results] is the most important thing for us, because it stops us from becoming crowded and becoming an infection spread risk.”* **P3**

Another key focus for prioritisation was the clinical areas where unplanned attendance is the normal access route e.g. ED, acute admissions and maternity. Point of Care (POC) or rapid testing was thought to be key in managing patient flow at these areas, because patient numbers are inherently unpredictable. There was concern that POC testing may lead to slowed turnaround at peak demand times, as throughput was limited on these types of devices.

In the case of test being restricted by lack of reagents or assays, it was agreed that testing ‘barn door’ clinically diagnosed cases of COVID-19 was unlikely to be the most effective use. Clinicians would not change management of these patients based on a negative test, therefore there seems little point in doing it. Tests might be better deployed to resolve cases where there is uncertainty.

*“So if they have got every single sign to suggest that they have got coronavirus, what is the test adding? Is it really adding anything? Probably not.”* **P13**

#### Characteristics of tests

From the test case priorities discussed in Table 2, Clinicians agreed that false negatives should be minimised, as these have the greatest detrimental effect on infection control measures. Additionally, sensitivity was reported as being most important in areas where there could be an infection risk to staff or where the outcomes for a positive patient were significantly worse i.e. aerosol generating procedures or thoracic surgery. At a minimum testing should be at least as sensitive as current tests. Rapid testing by participants was seen as necessary to facilitate patient flow through emergency and acute pathways.

*“I don't see a way forward without good testing, and there have been people said it from day one … and we abandoned it a certain early stage. But I don't see a way forward without rapid and repeated testing, and I think we need more and more of it this winter. That's all I can really say about that.”* **P5**

There were two main approaches to the potential placement of rapid tests: those who favoured the majority of testing being conducted in the central laboratory and those who thought more POC testing would need to be more widely deployed throughout the hospital. Those clinicians, who had an in-house laboratory generally thought that POC testing, whilst potentially useful in the ED, would show the greatest value by being placed in the laboratory.

*“Having had problems [with flu POC testing in ED], I’d much rather add a couple of hours on and have it done properly by someone who will record what happened.”* **P2**

Participants from smaller hospitals however, especially those who relied on an off-site central/regional lab, believed that Rapid/POC testing in the ED itself was vital to the next winter season.

*“We definitely need to have testing here. So, point of care. If it's not testing in ED by the nurses as it used to be before for Flu, definitely on site. So, either short point of care or the lab here on site to have the result in a couple of hours, or definitely the same day.”* **P9**

Few clinicians commented on the specific targets of the reference PCR or the individual platforms used within the trusts, those that commented on specific targets tended to be either be microbiologists or directly linked to the setup of RT-PCR testing within their laboratories. Clinicians did however comment on the wide range of platforms used for amplification, the most common being through the Public Health England RT-PCR Test, the Cepheid [17], or the SAMBA II [18] platforms. It should be noted however that a direct comparison between trusts, with regards to sensitivities and specificities of the tests, and their subsequent positive and negative predictive values cannot be made without further investigation into the specific platforms, standards and targets used and details of patient populations.

### Lessons learned

Good communication and dissemination of knowledge had been vital during a rapidly changing pandemic such as this. Examples of positive changes in communication were increased speed of information sharing e.g. via social media, breaking down of disciplinary silos, and moving patient consultations to phone or video consultation. Problems with communications were new guidance from central government being released on Friday afternoons, and the speed at which changes were required.

*“I think some bits worked reasonably well. I think the information and the speed of information sharing, often informally, was very helpful in terms of chest x-ray/CT appearances and clinical appearance of the disease.”* **P14**

Many were pleased with how much the situation had increased rate of service development and innovation within the NHS, the normal bureaucracy of change was minimised and hoped that it could continue. Clinicians also noted the increased adoption of new technology to facilitate communication. The standard NHS models for operating at 100% capacity, using just in time procurement or time limited incident response planning were not appropriate for pandemic situations. The NHS required greater flexibility to be able to provide a sustainable response to this.

*“For the first time in the NHS we really saw very few barriers between individuals and departments, and also the organisation. Things got ratified and moved around quickly. Some us believe we could have this all the time.”* **P12**

*“...we had a massive uptake in that we've got these kind of hands free badges that...you can call each other. And that was really helpful under PPE... So I think it was a good time, in terms of getting some transformation done.”* **P15**

### Feedback from PPIE panel

The research was well received by the panel who presented alternative views as to whether mass testing, including outpatients, is beneficial when the sensitivity of the current tests is reportedly so low. Some felt that the reported sensitivity of 70% was beneficial, whilst others felt it would undermine the confidence of the tests with the public by having 30% false negatives: and that any test would need to be a higher sensitivity before being rolled out for mass testing.

Further highlighted by the panel were the potential risks to testing patients in ED as, whilst it would be desirable to test them, the potential for the ED route to be seen as a community testing pathway by the public remained quite high. The panel therefore queried whether this would be effective given that the current system is pushing laboratory capacity to its limit.

## Discussion

To identify and characterise care pathways for patients with suspected or confirmed COVID-19 in UK hospitals, 16 clinicians working across 10 specialties, from 12 different NHS Trusts (including Trusts with no onsite laboratory facilities) were interviewed. We selected these Trusts to cover a wide geographical area and differentiation of needs depending on the different resources and facilities available, allowing us to obtain a comprehensive overview of the national approach.

This has been the first project of its kind in the UK and has been planned and delivered at pace to inform and support NICE in their early economic modelling of hospital testing strategy and resource utilisation. Previous published care pathway analysis for COVID-19 focused on pathways in individual hospitals, one in Rome, Italy [19] and one in New York, US [20]. This work instead took a different approach with a focus on drawing a broad, high-level picture of the pathway across the UK, whilst describing the differences across hospitals descriptively. This pathway was useful to inform economic modelling from a national perspective, as well as the study design of clinical evaluations of new diagnostics and POC tests with the CONDOR platform (https://www.condor-platform.org/).

### The prioritization of rapid testing

From the interviews, it emerged that COVID-19 testing is essential for infection prevention and control more than patient stratification for therapy. There was a higher pre-test probability of COVID-19 during the summer of 2020, due to the seasonal patterns often observed with flu and rhinovirus [21, 22]. Clinicians expressed concern that using clinical diagnosis alone during the winter season would pose a significant risk to both patients management and infection prevention as a whole, due in part to the likely circulation of other respiratory illnesses with similar symptoms [21, 22].

The main differences among hospitals’ strategies were due to real-estate constraints and resources, in particular the number of side bays, isolation rooms and single patient occupancy rooms, and the availability of rapid laboratory and testing facilities. Some interviewees reported having no onsite laboratory at their hospitals, instead relying on centralised services, a situation which may prolong turnaround times and lead to disjointed infection control systems [23]. In hospitals with limited estate it was difficult to efficiently manage patients whilst limiting the risk of nosocomial spread, since they relied on Yellow zones (often merged with Red zones). The Yellow zones, where patients with an uncertain diagnosis were waiting for test results, instead, should be minimized to limit virus spread between COVID-19 to non-COVID-19 patients. These cases are where rapid/POC testing may be beneficial, although throughput of samples will need to be large. There is also likely to be a limit on the number of tests available for POC due to lack of devices and/or reagents to carry out the tests. If test results take days to be reported, it has been shown that the longer people share a closed environment with a symptomatic COVID-19 infected person, the higher the risk of spread to the other people in the room. A UK based study identified that 89% of nosocomial cases in their hospital had shared a room with a SARS-CoV-2 PCR positive individual [24]. Thus, priority for providing rapid/POC testing in UK at hospital admission should be for these hospitals with limited numbers of isolation rooms and limited access laboratory testing. Furthermore, since the use of the Yellow zones should be minimized, their number should change adaptively depending on COVID-19 prevalence, as well as prevalence of respiratory conditions and demand on theatres and other essential procedures. Additionally, to allow for a flexible approach to zoning, it is important to have regular testing of staff to prevent asymptomatic or pre-symptomatic being incorrectly deployed into a green ward as the zones change [25].

The priority use cases for POC testing should instead be based on urgency of treatment and intervention, therefore in patients requiring emergency surgery, including caesarean section, especially if in need of general anaesthetic [26]. Rapid testing could assist surgical teams in their approach to the care of individual patients, for example when planning aerosol generating procedures or determining the appropriate level of PPE. Indeed, the use of the full PPE requires additional time and is uncomfortable for the surgeon when performing accurate procedures, potentially affecting patient outcomes [27]. The in-depth cleaning required after surgery of a COVID-19 positive patient (or of unknown status) limits the number of surgeries that the service can deliver in a day; therefore, using rapid testing for patients who required surgery would also support the high demand for theatres. It could also save resources by ensuring that patients who need access to the hyper-green areas of the hospital (i.e. where surgery happens), are triaged closer to their surgery date. This can reduce the risk that they have contracted SARS-CoV-2 between screening and admission.

### The rationing of multiplex tests

Since testing everyone at admission seems an inefficient strategy for large hospitals when the ED demand will rise in the winter months, the number of tests done should depend on prevalence and pre-test probability. Unless multiplex testing will be available, testing for COVID-19 only in patients with a high pre-test probability for COVID-19 is unlikely to change patient management, so resources might be better preserved in these situations. Multiplex testing seems more useful in hospitals where a flexible real-estate is available, allowing patients to be allocated in different wards depending on their differential diagnosis. If these facilities are not present in the hospital, multiplex testing may have the greatest advantage in frail and/or older patients where multiple sampling may be particularly uncomfortable and stressful. Due to the risk of bacterial co-infection with COVID-19, empiric broad spectrum antibiotics use has been widespread [28], and the provision of additional diagnostic information by multiplex testing could be used to improve antibiotic stewardship. A disadvantage of these tests (and of many POC tests for influenza) is that the presence of virus causes biosafety concerns, and the risk of missing some false negatives for COVID-19 after being (falsely) reassured of a positive result in another respiratory condition. Indeed, co-infection with influenza is possible in these patients during the winter season and should not be underestimated, with risk of death 5.92 times higher for co-infected individuals [29].

### Innovation in the NHS during the pandemic

A positive aspect of the pandemic was the reactivity of the system to the need and urgency. Extraordinary measures were quickly adopted, and this supported innovation and renovation within the NHS at an unusually fast pace. This approach was common also in other countries, especially the diffusion of remote clinics and medical research [30]. The rapidity in adopting innovation in a country where uptake has been notoriously very slow [31] has been recognised as a positive consequence by the interviewees, but it could have its drawbacks. For example, the authors noticed that some clinicians reported the clinical use of POC tests that at the time did not have strong and robust evidence of (in-context) diagnostic accuracy [32, 33]. Since the important role that tests had in the care pathway of fragile and very ill individual, we need to be careful that tests are used only once their performance has been carefully evaluated [34]. A test that does not work can be very harmful and much worse than no test at all, especially when relied upon for infection prevention.

### Limitations

We developed the topic guide and delivered the interviews at pace to maximize the support and advice we could offer to UK modellers and decision makers (NICE, for example). The authors are experienced in care pathway analysis and had been working on the COVID-19 pandemic from the very early stages of the disease, in close collaboration with numerous clinical experts from around UK, particularly through the CONDOR initiative, thus the quality of the interviews was high even though developed at pace.

A key limitation to the methodology is the systematic bias which arises in following a structured interview approach. By necessity, the questions were guided towards specific topics of interest, therefore limiting the scope of potential answers [35]. However, the interviews were also open ended which allowed for some degree of free flowing conversation, particularly if the interviewee had strong feelings to express. A completely unstructured approach would have likely been a severe dilution of the main narrative across the interviews.

From the candidates who were initially canvassed, all but one of those who dropped out represented a specialty already covered by the interviews, with only Ambulatory Care not being captured. Additionally, they all held similar roles and responsibilities to those of the final group interviewed, with three clinicians also being from a previously interviewed trust. Therefore, whilst the sample size is small, due in part to difficulties of recruitment at the time, the large drop out rate was not felt to have hampered the coverage of specialisms significantly. Furthermore, whilst the sample size was not large, this was in part due to reaching saturation of information quickly. This happened despite interviewing a varied sample of people in terms of expertise, location of hospitals and their estate’s characteristics. Recommendations for COVID-19 pathways were provided centrally, so the differences between hospitals were relatively minor [36, 37]. This might explain the limited sample needed to reach saturation.

## Conclusions and next steps

The NHS reacted quickly to the pandemic, designing care pathways appropriate to their estates and facilities. Differences in patient management between hospitals were largely due to the time required to have diagnostic results from standard laboratory RT-PCR testing. Pathways were suboptimal in hospitals where the time to results was greater than 24 hrs. Most clinicians expressed the need for quicker diagnostics, in particular POC testing, in hospitals where access to rapid laboratory testing was absent. This is especially urgent now that the winter has arrived and respiratory diseases with similar symptomology to COVID-19 may soon be in circulation.

Our work identified and described the pathway used in NHS hospitals and informed the early economic modelling underway by NICE and the study design of the CONDOR clinical diagnostic accuracy studies in hospitals. We identified the hospitals and patient groups to prioritize for implementation of the rapid diagnostics and POC tests once robust evaluations of their in-context diagnostic accuracy are concluded. The winter season brings additional complexity to identification of patients with COVID-19, due to additional respiratory viruses on a patient’s differential diagnosis. Tests whose accuracy have not been estimated in the hands of the end user, in the correct patient population, and in the correct clinical context could be more harmful than helpful. Thus, robust evaluations are essential even though the urgency of the situation requires speedy evaluations.

## Supplementary Information

**Additional file 1.** COVID-19 Secondary Care - Original Protocol

**Additional file 2.** COVID-19 Secondary Care - Topic Guide

**Additional file 3.** COVID-19 Secondary Care - SRQR Checklist

**Additional file 4.** Members of the CONDOR Steering Group

## Data Availability

Data collected for this study will be made available to researchers who provide a methodologically sound research proposal to assist with the aims in the approved proposal. Data will be available from the time of publication of this article in print. Proposals should be directed to the corresponding author.
