# Supplementary material for: Care pathway and prioritization of rapid testing for COVID-19 in UK hospitals: a qualitative evaluation
Source: BMC Health Serv Res. 2021 May 31;21:532. doi: 10.1186/s12913-021-06460-x (PMC8165513; doi:10.1186/s12913-021-06460-x)
Supplement: Supplementary file 1 — Additional file 1. COVID-19 Secondary Care - Original Protocol [file 12913_2021_6460_MOESM1_ESM.pdf]

National Institute for Health Research

Newcastle In Vitro Diagnostics Co-operative

Care pathway analysis for COVID-19 testing in hospitals and in the community

Authors: Sara Graziadio, Tim Hicks, Joy Allen and Ashley Price

Principal Investigator's name(s): Ashley Price

Signature:

Date: 16/06/2020

The Newcastle upon Tyne Hospitals NHS Foundation Trust
Newcastle University
The Newcastle MIC is funded by the NIHR and delivered in partnership with Newcastle upon Tyne Hospitals NHS Foundation Trust and Newcastle University

Table of Contents

1 Investigators 2
2 Background & Rationale 4
2.1 Clinical problem 4
2.2 Definition 4
2.3 Care pathway 4
2.4 Clinical need and evidence gaps 5
3 Aims 5
4 Design of the evaluation 5
4.1 Overview of design 5
4.2 Methods: reviews and interviews 5
4.3 Stakeholder Identification 6
4.4 Eligibility Criteria 6
4.5 Sample size considerations 7
4.6 Participant recruitment 7
4.7 Consent 7
4.8 Ethical approval 7
4.9 Data management 8

1 Investigators

Dr Ashley Price, MD
Consultant in Infectious Diseases
Royal Victoria Hospital
Richardson Rd,
Newcastle upon Tyne
NE1 4LP

Dr Joy Allen PhD
Senior IVD Evaluation Methodologist
NIHR Newcastle In Vitro Diagnostics Co-operative
Rm M2.088 William Leech Building
Medical School,
Newcastle University,
Framlington Place
Newcastle upon Tyne
NE2 4HH

Dr Sara Graziadio PhD
Senior IVD Evaluation Methodologist
NIHR Newcastle In Vitro Diagnostics Co-operative
Rm M2.088 William Leech Building
Medical School,
Newcastle University,
Framlington Place
Newcastle upon Tyne
NE2 4HH

Amanda Winter
IVD Evaluation Methodologist
NIHR Newcastle In Vitro Diagnostics Co-operative
Rm M2.088 William Leech Building
Medical School,
Newcastle University,
Framlington Place
Newcastle upon Tyne
NE2 4HH
0191 2083708

Tim Hicks
IVD Evaluation Methodologist
NIHR Newcastle In Vitro Diagnostics Co-operative
Rm M2.088 William Leech Building

Medical School,
Newcastle University,
Framlington Place
Newcastle upon Tyne
NE2 4HH
0191 2083708

2 Background & Rationale

2.1 Clinical problem

The World Health Organization (WHO) was informed of cases of pneumonia of unknown microbial aetiology associated with Wuhan City, Hubei Province, China on 31 December 2019. The WHO later announced that a novel coronavirus had been detected in samples taken from these patients. Since then, the epidemic has escalated and rapidly spread around the world, with the WHO first declaring a public health emergency of international concern on 30 January 2020, and then formally declaring it a pandemic on 11 March 2020. The condition has been given the official name of coronavirus disease 2019 (COVID-19). Clinical trials and investigations to learn more about the virus, its origin, how it affects humans, and its management are ongoing.

2.2 Definition

COVID-19 is a potentially severe acute respiratory infection caused by the novel coronavirus severe acute respiratory syndrome coronavirus 2 (SARS-CoV-2). The clinical presentation is generally that of a respiratory infection with a symptom severity ranging from a mild common cold-like illness, to a severe viral pneumonia leading to acute respiratory distress syndrome that is potentially fatal. Characteristic symptoms include fever, cough, and dyspnoea, although some patients may be asymptomatic. Complications of severe disease include, but are not limited to, multi-organ failure, septic shock, and blood clots.

2.3 Care pathway

COVID-19 care pathways were established at local, regional, and national levels for people with suspected or confirmed COVID-19. Guidelines and pathways changed during the pandemic reflecting the infection rate. Also their uptake changed, mainly depending on the availability of tests, Personal Protective Equipment and isolation rooms in each setting.
In general, recommendations were that patients were screened at the first point of contact within the health system based on case definitions and assessment of symptoms. Advise was to immediately isolate patients who were suspected or confirmed COVID-19 cases implementing local infection prevention and control procedures. Severity of disease was assessed with standardised triage tools. Guidelines recommended that suspected cases should remain in the pathway until proven negative.

2.4 Clinical need and evidence gaps

Early recognition and rapid diagnosis are essential to prevent transmission and provide supportive care in a timely manner. Diagnostic tests play a major role in this context but evidence for their performance and utility are still lacking. It is also unknown what is the optimal role for each test (laboratory or point of care; antibody or molecular) in the each setting and at what point of the pathway each test should be included for the evaluation and, later, for implementation.

3 Aims

This project will build on the work determining current and previous care pathways in hospital and care home settings (see protocol: v03 dated 19/06/20), specifically focussed in primary care settings, particularly with GPs.

The aim is to understand the changes in Clinical Guidance and diagnostic pathways throughout the pandemic, focusing on pathway development over time and other socio-economic factors which may have influenced the effectiveness of the testing strategy and subsequent pathways.

The ultimate aim of this project is to gain a broad understanding of what interventions/changes worked during the initial wave in order to inform NICE modelling to optimize diagnostic testing strategy, and potentially hospitals and DHSC to plan implementation of new strategies if a second wave of the pandemic occurs.

4 Design of the evaluation

4.1 Overview of design

Methodologists within the MICs will hold interviews with clinicians who had significant involvement in diagnosis and management of patients with COVID in UK NHS hospitals, GP surgeries and care homes. The clinical interviews will be semi-structured, facilitating a broad range of interviewee perspectives and responses, whilst also allowing the interviewers to expand their questioning on themes of interest (i.e. those pertinent to the interview aims).

4.2 Methods: reviews and interviews

- Review of current and past guidance (NICE, DoH, Local Trust Policies etc.) alongside wider socio-economic changes both regional and from the wider government (lockdown implementation, restrictions in travel, social distancing etc.)

- Process mapping of the different pathways and their monthly changes from March 2020 to July 2020 to understand the patient flow and the utilisation of the different testing pathways along with any barriers to implementation or use.

- Pathway Presentation as part of a “Living Pathway Analysis” in which the pathway changes over time are documented and highlighted.

These findings will inform a small number of clinical expert interviews to help consolidate our understanding of the current pathway and clinical need in COVID-19 identification, and allow the pathway, how the guidelines were implemented and changed over time, and any possible bottlenecks/problems within the system.

Thematic analysis of the interviews will be used to explore, categorise, and document the opinions and information obtained in the interviews.

4.3 Stakeholder Identification

The investigators part of the CONDOR initiative will assist the MIC methodologists to identify potential interviewees from within their clinical network and will introduce them to the researchers. MIC methodologists may also identify potential interviewees through their own networks as appropriate

Interviews will be sought from social and healthcare professionals in the following disciplines or specialisms or roles:

- Medical Microbiology
- Laboratory managers & biomedical scientists
- Infection Control
- Infectious Diseases
- General Practitioners
- General practice managers
- Care home managers

Interviews will be sought from a number of geographical areas, in order to gain the best representation of practice across the country. The relatively small number of interviews will restrict the extent to which this is achievable.

4.4 Eligibility Criteria

To be eligible for inclusion the participants must have relevant expertise and experience of the management and diagnosis of COVID-19 patients in hospitals and GP surgeries, or in the management of healthcare and social care facilities. Additionally the participants must be fluent English speakers as the output of the evaluation will be developed from the recorded interviews.

4.5 Sample size considerations

Due to the exploratory nature of the work, we have opted for a small and carefully selected group of clinical experts and managers to provide the relevant clinical picture. The maximum sample size for interviews is 10. This number is based on reaching information saturation and is estimated from previous interview-orientated studies performed by the team.

4.6 Participant recruitment

Potential interviewees will be approached via email, in which they will be invited to be interviewed by telephone/Skype.

4.7 Consent

All potential participants will be sent a Participant Information Sheet by email prior to enrolling in the evaluation. The interview will take around one hour of the interviewee's time. If they agree to participate, where possible a face-to-face meeting or telephone appointment with the researchers will be arranged.

As this work is service evaluation, consent is not required. However, the MIC methodologist will confirm verbally before beginning the interview that the participant still wishes to participate and that they are happy for the interview to be recorded. The audio recording will be used as a back-up of the interview, and for verification of the details of the discussion, in case the notes taken during the interview are not extensive. Additional informal permissions will be sought to report any anonymised quotes, and invite the participant to sense check the findings of the analysis (in particular the care pathways developed as a result of these interviews and literature reviewing) and to take part in future studies.

They will be asked to confirm their understanding that no patient identifiable information should be provided, that the researcher accepts no liability for accidental disclosure. If accidental disclosure of patient identifiable information happens, this information will not be transcribed or published for any purpose, but the audio recording (if consented to) may remain.

The participants may withdraw at any stage of the process and can request that all information collected up to that point is destroyed.

4.8 Ethical approval

There will be no involvement with patients, or samples taken for tests. Any information from the evaluation will be anonymized and unable to be attributed to any individual when used in reports.

Ethical approval by the HTA is not required. The evaluation will be recorded as a service evaluation by the Joint Research Office of Newcastle University, and the Newcastle upon Tyne Hospitals NHS Foundation Trust and a copy of this protocol retained for their records.

4.9 Data management

Data collected from each interview will consist of handwritten notes, and if the interviewee agrees, an audio recording. This will be carried out using online video conferencing (e.g. Zoom). At the earliest opportunity following the interview, a researcher will transfer the recording to a password-protected computer. All other outputs arising from the interview will be stored, and processed, in the same secure manner.
