# Supplementary material for: Care pathway and prioritization of rapid testing for COVID-19 in UK hospitals: a qualitative evaluation
Source: BMC Health Serv Res. 2021 May 31;21:532. doi: 10.1186/s12913-021-06460-x (PMC8165513; doi:10.1186/s12913-021-06460-x)
Supplement: Supplementary file 2 — Additional file 2. COVID-19 Secondary Care - Topic Guide [file 12913_2021_6460_MOESM2_ESM.pdf]

National Institute for Health Research

Newcastle In-Vitro Diagnostics Co-operative
(Newcastle MIC)

Topic guide - COVID-19 Testing in Secondary Care

Care pathway analysis for COVID-19 testing within Secondary Care Settings

Introduction

"Thank you for taking time out of your busy schedule to participate in this interview."

Check they have received the necessary documents and that they are happy to be recorded.

"We have a semi-structured interview script to take you through, but I think it would be useful if we first introduced ourselves to you, tell you a bit about the type of work we do - then see if you have any questions - then move onto the interview, does that sound OK?"

"We work for the Newcastle MIC, which stands for the MedTech In Vitro Diagnostics Cooperative."

"We have expertise in the evaluation of diagnostic devices and tests, and our overall aim is to support appropriate evidence generation.

"We are independent evaluators, funded by the NIHR. We are currently part of the national CONDOR platform, and this work will potentially inform target product profiles for MHRA, economic modelling at NICE as well as supporting diagnostic companies and CONDOR investigators to make decisions about which devices and tests to further develop and evaluate."

"To help us with this work, we would like to (first) understand current practice for the diagnosis and management of patients with respiratory infections, primarily focusing on those where there is a suspicion of COVID-19."

"Do you have any questions for us, so far?"

Interview

<u>Background Questions</u>

1. **Where do you work in UK?**
2. **In which Trust do you work?**
3. **Is there an outbreak currently in your Trust? Or recently?**
4. **What is your role within the hospital?**
5. **In which section(s) of the hospital do you work?**
6. **Were you relocated during the pandemic? If so, where?**
7. **How many years' experience do you have post-qualification?**

NHS National Institute for Health Research

Current Pathway Questions

We sent ahead of the interview a draft of the pathway for admission of patients with potential COVID-19 across acute secondary care.

Please comment on:

- *How this aligns with your experience? Please indicate anywhere where your Trust's pathway differs from the pathway presented.*
- *How the admission pathway differs for different aspects of secondary care e.g. emergency admission, surgery, minor injuries? Any key similarities/differences in terms of testing strategies/isolation/PPE use?*
- *Do you have a Medical Assessment Unit where you triage patients arrived from GP referrals? Where would patients referred by 111 arrive? Are these admission pathways different from the one presented in our flow diagram in terms of testing/isolation/PPE use?*

What tests are currently in use to detect COVID-19 within Secondary Care?

- *Who are you testing exactly?*
- *What tests are you using? Lab tests/POCTs?*
- *Do you think in the winter the number of people you would like to test will increase? If so, how much?*
- *How confident in the results derived from investigations/testing procedures are you?*
- *When samples are taken where is this done? E.g. side rooms*
- *What samples exactly are taken?*
- *Who is involved in the sampling process (number of people, their training and background)?*
- *Are they trained specifically in taking samples for COVID testing? E.g. nasopharyngeal swabs*
- *Do you feel the current tests meet the requirements in a Secondary Care setting? I.e. availability, ease of use, training required, patient discomfort (not diagnostic accuracy at this point)*
- *What is the average turnaround time from testing to receiving results?*
- *Who receives results and what procedures are in place to action them?*
- *What are the outcomes of these actions in terms of benefits and harms of these actions?*
- *Do you isolate/cohort patients with positive tests? How is the organization that you set up around isolation? Do you envisage problems with this policy as we see more respiratory pathogens in the winter?*
- *Will the present pathway differ in the winter?*

In your opinion, where are the problems within current decision making and testing?

- *How might COVID testing or multiplex testing could help resolve these issues?*
- *Which are the roles for testing/populations to prioritize, ie where there is a higher unmet clinical need for accurate testing and where for quick testing?*

Do you think the pathway will be need to be adapted in future, e.g. if there was a second wave, or in the winter? If so, how?

Temporal Pathway Questions

There have been a number of changes made to the pathway over the course of the pandemic, please describe how each of the following three changes have impacted your work area:

1. *Introduction of staff testing*
2. *Screening for COVID-19 of all patients on admission*
3. *Separation of hospital into red, amber and green zones for cohorting.*

Are there any other changes which have significantly impacted your practice?

What can be learned from these changes to inform future practice?

- Any implementation challenges?
- Anything that can help us make better use of diagnostics in the future (for next peak of the pandemic and/or for the flu season)?

Pathway Improvement Questions

Let's imagine the possible scenario where there aren't enough reagents/POCTs to test everyone with potential COVID-19 at the hospital doors in the next winter season.
Who do you think should be prioritized for testing in a hospital setting (with lab testing and POCTs)?

Please identify the 8 most important populations to prioritize with a Y and if there is any population in this list not to prioritize with a N. For all the populations except the N, which ones would need a POCT (10-40 min TAT) instead of an RT-PCR (6-36 hours TAT)?

Only for the POCT, what would be the minimum acceptable percentage of COVID patients you could accept to miss and the percentage of non-COVID patients you could accept to incorrectly diagnose as COVID?

NHS National Institute for Health Research

				Priority (Y or N)	POCT for COVID needed? (Y/N/not sure)	Acceptable perc. of missed COVIDs by POCT	Acceptable perc. of non-COVIDs mis-diagnosed by POCT
Symptomatic* for COVID (severe**)	high risk***	Patients	requiring any urgent surgery				
			requiring hearth and abdominal surgery				
			requiring transplantation				
			requiring any other not-urgent surgery				
			requiring aerosol procedures				
			requiring chemotherapy				
			requiring dialysis				
			requiring hospital admission				
			requiring assessment in A&E or MAU				
		Staff	(for diagnosis) influencing return to work				
symptomatic for COVID (severe)	low risk	Patients	requiring any urgent surgery				
			requiring hearth and abdominal surgery				
			requiring transplantation				
			requiring any other not-urgent surgery				
			requiring aerosol procedures				
			requiring chemotherapy				
			requiring dialysis				
			requiring hospital admission				
			requiring assessment in A&E or MAU				
		Staff	(for diagnosis) influencing return to work				
symptomatic for COVID (mild)	high risk	Patients	requiring any urgent surgery				
			requiring hearth and abdominal surgery				
			requiring transplantation				
			requiring any other not-urgent surgery				
			requiring aerosol procedures				
			requiring chemotherapy				
			requiring dialysis				
			requiring hospital admission				
			requiring assessment in A&E or MAU				
		Staff	(for diagnosis) influencing return to work				
symptomatic for COVID (mild)	low risk	Patients	requiring any urgent surgery				
			requiring hearth and abdominal surgery				
			requiring transplantation				
			requiring any other not-urgent surgery				
			requiring aerosol procedures				
			requiring chemotherapy				
			requiring dialysis				
			requiring hospital admission				
			requiring assessment in A&E or MAU				
		Staff	(for diagnosis) influencing return to work				
asymptomatic for COVID	high risk	Patients	requiring any urgent surgery				
			requiring hearth and abdominal surgery				
			requiring transplantation				
			requiring any other not-urgent surgery				
			requiring aerosol procedures				
			requiring chemotherapy				
			requiring dialysis				
			requiring hospital admission				
			requiring assessment in A&E or MAU				
		Staff	going to work/PPE use				
asymptomatic for COVID	low risk	Patients	requiring any urgent surgery				
			requiring hearth and abdominal surgery				
			requiring transplantation				
			requiring any other not-urgent surgery				
			requiring aerosol procedures				
			requiring chemotherapy				
			requiring dialysis				
			requiring hospital admission				
			requiring assessment in A&E or MAU				
		Staff	going to work/PPE use				

*'Symptomatic for COVID' is defined as: Persistent cough and/or fever and/or anosmia and/or myalgia

**'Severe' is defined as: anyone requiring admission with oxygen requirement and/or infiltrates on CXR[1]

***'High risk' is defined as: >65, BAME, diabetic, residents of care/nursing homes, pregnant[2]

Would you agree with the definition provided for symptomatic patients/severe patients/high risk patients in the list above?

Please explain why you prioritised those patient groups, the need for POCT, sensitivity and specificity levels. What is the most important role in the hospital for POCT in those patient groups?

Should we have some other patient groups to be prioritized? Which ones?

Multiplex tests are being developed to detect SARS-CoV-2 alongside other respiratory pathogens.

- *Would you see a value in these tests compared to a COVID-only test? Which one(s)?*
- *In which settings and populations in particular? Link to above list*
- *Which respiratory pathogens should be included in the multiplex?*
- *How would you usually distinguish COVID-19 from other respiratory conditions?*
- *What evidence would you like to see to support their use in this way?*
- ***If they are involved/experts in evaluations** of tests, also ask:*
  What do you think the main challenges are for these clinical evaluations?

Do you think an antibody tests could be useful in a hospital setting? If so, how?

- *in which context? (patients, use in the pathway) any patient populations above.*
- *What would be the minimum level of sens and spec required to be useful in the context you previously described?*
- *What would you like to see in terms of evidence to support their use?*

For the patient populations prioritised above, what might the role for combination testing be (e.g. POCT + lab test, Viral + antibody)?

Is there a group of patients who would require frequent testing? Who? How often do you think testing would be useful and feasible?

References

1 Drew DA, Nguyen LH, Steves CJ, et al. Rapid implementation of mobile technology for real-time epidemiology of COVID-19. *Science*. 2020;368(6497):1362-1367. doi:10.1126/science.abc0473

2 Cabinet Office Guidance: Staying alert and safe (social distancing) - Updated 3 July 2020 https://www.gov.uk/government/publications/staying-alert-and-safe-social-distancing/staying-alert-and-safe-social-distancing-after-4-july#clinically-vulnerable-people
