# Supplementary material for: Care pathway and prioritization of rapid testing for COVID-19 in UK hospitals: a qualitative evaluation
Source: BMC Health Serv Res. 2021 May 31;21:532. doi: 10.1186/s12913-021-06460-x (PMC8165513; doi:10.1186/s12913-021-06460-x)
Supplement: Supplementary file 4 — Additional file 4. Members of the CONDOR Steering Group [file 12913_2021_6460_MOESM4_ESM.docx]

**S.4 – Members of the CONDOR Steering Group**

Julian Braybrook^8^, Prof Peter Buckle^9^, Prof Paul Dark^10^, Kerrie Davies^11^, Eloise Cook^12^, Prof Adam Gordon^13^, Anna Halstead^1^, Prof Gail Hayward^14^, Prof Dan Lasserson^15^, Andrew Lewington^16^, Brian Nicholson^14^, Prof Rafael Perera-Salazar^14^, Prof John Simpson^1^, Philip Turner^14^, Graham Prestwich^17^, Charles Reynard^6^, Beverley Riley^16^, Valerie Tate^18^, Prof Mark Wilcox^16^, Colette Inkson^19^

^8^UK National Measurement Laboratory, Middlesex, United Kingdom

^9^Faculty of Medicine, Department of Surgery & Cancer, Imperial College London, London, United Kingdom

^10^Division of Infection, Immunity and Respiratory Medicine, The University of Manchester, Oxford Road, Manchester M13 9PL, United Kingdom

^11^Healthcare Associated Infections Research Group, Leeds Teaching Hospitals NHS Trust and University of Leeds, Leeds, United Kingdom.

^12^The University of Manchester, Oxford Road, Manchester M13 9PL, United Kingdom

^13^Faculty of Medicine & Health Sciences, University of Nottingham, Nottingham, United Kingdom.

^14^Nuffield Department of Primary Care Health Sciences, University of Oxford, Oxford, United Kingdom

^15^Institute of Applied Health Research, University of Birmingham, Birmingham, West Midlands, United Kingdom.

^16^National Institute for Health Research (NIHR) In-Vitro Diagnostic Co-operative, Leeds, United Kingdom

^17^Yorkshire and Humber Academic Health Science Network, Wakefield, United Kingdom

^18^Oxford Academic Health Science Network, Oxford, United Kingdom

^19^Division of Cell Matrix Biology & Regenerative Medicine, The University of Manchester, Oxford Road, Manchester M13 9PL, United Kingdom
